# Significance and Therapeutic Value of miRNAs in Embryonal Neural Tumors

**DOI:** 10.3390/molecules19055821

**Published:** 2014-05-06

**Authors:** Tarek Shalaby, Giulio Fiaschetti, Martin Baumgartner, Michael A. Grotzer

**Affiliations:** Department of Oncology, University Children’s Hospital of Zurich, Zurich 8008, Switzerland

**Keywords:** embryonal tumors, microRNAs, medulloblastoma, sPNET, AT/RT, neuroblastoma

## Abstract

Embryonal tumors of the nervous system are the leading cause of childhood cancer-related morbidity and mortality. Medulloblastoma, supratentorial primitive neuroectodermal tumors, atypical teratoid/rhabdoid tumor and neuroblastoma account for more than 20% of childhood malignancies and typify the current neural embryonal tumor model in pediatric oncology. Mechanisms driving the formation of these tumors point towards impaired differentiation of neuronal and neuron-associated cells during the development of the nervous system as an important factor. The importance of microRNAs (miRNAs) for proper embryonic cell function has been confirmed and their aberrant expressions have been linked to tumor development. The role of miRNAs in controlling essential regulators of key pathways implicated in tumor development makes their use in diagnostics a powerful tool to be used for early detection of cancer, risk assessment and prognosis, as well as for the design of innovative therapeutic strategies. In this review we focus on the significance of miRNAs involved in the biology of embryonal neural tumors, delineate their clinical significance and discuss their potential as a novel therapeutic target.

## 1. Introduction

Embryonal tumors refer to a broad heterogeneous group of childhood malignancies that account for approximately 30% of childhood malignancies, including neuronal tumors of the central and peripheral nervous system, in addition to other rare subtypes, such as rhabdomyosarcomas, Wilms’ tumor, and retinoblastoma [1]. Advances in cancer biology research have increasingly linked pediatric neoplasms with disordered mechanisms of normal development, supporting the model of embryonal tumorigenesis, both processes involve self-renewal and precisely coordinated proliferation, differentiation and cell migration [2]. The significances of miRNA-mediated regulation of gene expression during early embryogenesis and those that were linked to tumorigenesis have been established in human embryonal stem cells [3,4,5,6,7,8,9,10,11,12,13,14,15,16,17,18,19,20,21]. Based on these sightings, specific miRNA genes responsible for the control of embryonal stem cell proliferation and differentiation have been identified, which were also found to contribute to the initiation and progression of cancer stem cells [22,23,24,25,26]. Although the role of miRNA function in embryonal tumor development is still not completely understood, it is becoming clear that miRNAs are essential regulators of many of the key pathways implicated in the pathogenesis and progression of pediatric nervous system tumors [27]. This review focuses on the current understanding of the role miRNAs play in embryonal tumor development and progression, and will discuss their potential as new therapeutic targets.

## 2. Embryonal Tumors of the Central and Peripheral Nervous System

Embryonal tumors differ fundamentally from adult onset cancers, both in their cell biology and their tissue environment. They develop and manifest early in childhood and are usually characterized by a high rate of mortality and treatment-related morbidity [28]. Embryonal CNS tumors are a collection of biologically heterogeneous lesions that share the tendency to disseminate throughout the nervous system via cerebrospinal fluid (CSF). Embryonal CNS tumors include medulloblastoma (a malignant invasive tumor of the cerebellum), CNS primitive neuroectodermal tumors (a heterogeneous group of tumors arising in the cerebral hemispheres, brain stem or spinal cord), and atypical teratoid/rhabdoid tumors (AT/RT; a highly malignant CNS tumor predominantly manifesting in young children) [29]. Neuroblastoma (NB)—the most common extra-cranial embryonal tumor of childhood—arises in the sympathetic nervous system. 

## 3. microRNAs

miRNAs are small non-coding RNAs that act as expression regulators of genes involved in fundamental cellular processes, such as development, differentiation, proliferation, survival and death [30]. Over the past few years, specific miRNAs have been reported to be implicated in various human cancers including brain tumors [9,10,11,12,13,14,15,16,17,18,19,20,21]. miRNAs were linked to tumorigenesis due to the fact that 50% of the miRNAs genes are located at genomic sites associated with cancer-specific chromosomal rearrangements as well as due to the proximity of their genes to chromosomal breakpoints [31,32]. It has been found that miRNAs have the ability, in the context of human cancer, to behave like oncogenes or tumor suppressors, a finding which has important implications for cancer etiology and diagnosis [33,34,35]. Both losses and gains of miRNA functions through deletions, amplifications and mutations in miRNA loci or epigenetic silencing can contribute to cancer development [36]. The frequent aberrant expression and functional implication of miRNAs in human cancers have lifted these small cellular components to the ranks of preferred drug targets that operate by a new mechanism of action.

## 4. miRNAs in Embryonic Cell Function and Tumor Development

Increasing evidence suggests that embryogenesis and tumorigenesis display common characteristics. Both processes involve self-renewal and proliferation, differentiation and cell migration. Based on these parallels, researchers have begun to examine the role of miRNAs in neural precursor development and cell fate determination in order to determine whether brain developmental pathways are linked to brain tumor etiology [37]. Numerous publications demonstrate the importance of the developmental effects that are related to the miRNA-mediated regulation of gene expression during early embryogenesis in different species including *C. elegans*, Drosophila, Zebrafish and mice [38,39,40,41,42,43,44,45,46,47]. In humans, experimental data shows that miRNAs control the fate of human ESCs during early development [39,48,49,50,51,52,53,54]. Human ESCs for example, express a unique microRNAs (miR-200c, 368, 154*, 371, 372, 373*, 373) [42] that might serve as molecular markers for the undifferentiated stem cells in early embryonic stage. The importance of miRNAs in the development of the mammalian nervous system was demonstrated in mouse mutants lacking the activity of Dicer, the type III RNAse that is necessary for miRNA biogenesis, which die at embryonic day 7, reviewed in [55]. Using zebrafish as a model, researchers discovered that specific miRNAs such as miR-92b, miR-124, miR-222, miR-218a, miR-26 and miR-29, miR-9 are expressed differentially between neural precursors and stem cells [56,57]. In differentiating embryonic stem cells of mammals, miRNAs that are specifically expressed or enriched in brain tissues, such as let-7a, miR-218, and miR-125, miR-200a, miR-200b, miR-429, miR138 and miR-141 with miR-124 and miR-9, were found to increase sharply in abundance during the transition from neuronal precursors to neurons [41,44,58,59,60,61,62,63,64,65,66,67]. Studying the expression pattern of miRNAs during mammalian brain development, Kim *et al.* [68] identified eight miRNAs (miR-103, -124a, -128, -323, -326, -329, -344, -192-2) to be temporally expressed in the rat cortex and cerebellum during neural differentiation and development [69]. In another study, using human embryonic cells that differentiate into neurons upon retinoic acid treatment, Sempere *et al.* showed that miR-9/9, -103-1, -124a, -124b, -128, -135, -156, -218 were co-ordinately induced during the neural differentiation process [60]. Some of these miRNAs are the same as the candidates Kim and colleagues showed to be developmentally regulated in rat brain corticogenesis, indicating important roles for these miRNAs in neuronal cell fate specification and differentiation. Remarkably some of these miRNAs were reported to be also active in neuron-developmental disorders [70].

The notion that cancer is fuelled by self-renewing stem cells is gaining prominence, and so is the idea that miRNA can direct cell fate. Yu F. *et al.* recently brought the two fields together by showing that a single miRNA molecule “miRNA let-7” can regulate stemness in breast cancer cells [22]. The important role of stem cells both in tissue and cancer development has driven much of the research into neural cancer stem cell biology. This research recently led to the identification of specific miRNA genes responsible for ESCs proliferation and differentiation and for the initiation and progression of cancer stem cells [22,23,24,25,26]. Silber *et al.* reported that miR-124 and miR-137 induce differentiation of neural stem cells and glioblastoma stem cells [25]. Desanomi *et al.* reported that miR-34 deficiency is involved in the self-renewal and survival of cancer stem cells and in cancer cells lacking functional p53, restoration of miR-34 was able to re-establish the tumor suppressing signaling pathway [26]. Amazingly, specific miRNAs such as miR-302 were recently found to be capable of reprogramming the cancer cells back into a pluriopotent embryonic stem cell-like state, which then could be induced into tissue-specific mature cells [71]. Together these reports suggest that miRNA expression is vital for normal as well as abnormal ECS development that can lead to cancer stem cell initiation, reviewed in [26].

Cancer stem cells have been identified, isolated and characterized recently in embryonal neural malignancies including MB [72,73,74,75,76]. With the knowledge that MB harbors cancer-initiating cells with stem cell properties, scientists are making a great effort to understand the involvement of aberrantly expressed miRNAs in embryonal cancer stem cells and to elucidate the mechanisms, which distinguish these cells from normal stem cells. Functional studies on miRNAs within the cancer stem cells of MB will be crucial for elucidating the mechanisms behind oncogenesis in these deadly malignancies. The gained insight might reveal novel therapeutic targets, whose inhibition could impact the cancer-initiating cells and thus greatly reduce the risk of cancer relapse.

## 5. Role of miRNAs in Embryonal Neuronal Tumors

### 5.1. Medulloblastoma

MB is the most common malignant tumor of the central nervous system and represents more than 20% of all pediatric brain tumors [77]. MB is an invasive embryonal tumor of the cerebellum with an inherent tendency to metastasize via the subarachnoidal space [29]. Five histological varieties are recognized in the 2007 WHO classification: classic, anaplastic, large cell anaplastic, desmoplastic/nodular variant and MB with extensive nodularity [78]. Associations have been made between histopathological subtype and clinical outcomes. Patients with classical tumors tend to have average clinical outcomes, while desmoplastic/nodular MBs are associated with improved survival [78,79]. In contrast, large cell and anaplastic histologies predict shorter survival [79,80]. Several groups around the world have demonstrated that MB is not one disease but comprises a collection of distinct molecular subgroups. The current consensus is that there are four core subgroups, which should be termed WNT, SHH, Group 3 and Group 4 [81,82,83,84]. Kool and colleagues performed recently a meta-analysis of all molecular and clinical data of 550 MBs brought together from seven independent studies [85]. All cases were analyzed by gene expression profiling and for validation purposes they compared the results of this meta-analysis with another large (402) MB cohort for which subgroup information was obtained by immuno-histochemistry. The results from both cohorts were highly similar and confirmed the existence of the four MB subgroups and showed how distinct the molecular subtypes are with respect to their transcriptome [85]. Linking the subgroups to clinical follow-up data demonstrated that WNT and SHH carry favorable outcomes while group 3 and 4 are associated with poor prognosis [81,82,84,86,87]. The best outcome was found for WNT tumors. Interestingly, metastatic disease at diagnosis is also significantly associated with non-WNT/SHH tumors and most strongly with group 3 tumors [81,82,84].

5.1.1. miRNAs Aberrantly Expressed in MB

Despite the intense investigation in the involvement of miRNAs in a variety of cancer types, knowledge of their role in MB pathogenesis has only recently started to build up. Pierson *et al.* [88] was the first to report the involvement of miRNA in MB. The authors found that the expression of miR-124a—a brain enriched miRNA preferentially expressed in mature neurons and in external granule cells of the cerebellum, which were reported to be cells of origin of MBs [44,65,67]—is significantly decreased in MB cells compared to normal cerebellum. Similar decreases were also observed in other brain tumors [25,69]. Interestingly restoration of miR-124 function inhibits MB cell proliferation, suggesting that it may act as a suppressor of tumor growth. In a computational-based approach to predict miR-124 target genes, Pierson *et al.* discovered and validated cyclin dependent kinase 6 (CDK6) that was identified as an adverse prognostic marker in MB and solute carrier family 16, member 1(SLC16A1) that may represent a novel therapeutic target for treatment of malignant MBs [89,90]. In a parallel but distinct study Ferretti *et al.* [27] used high-throughput screening to examine miRNA expression profiles in 34 patients with MB. Their investigation revealed a set of 78 miRNAs with altered expression in MB, compared to normal adult and fetal cerebellar samples. Amazingly the authors identified a unique set of four up-regulated miRNAs (let-7g, miR-19a, miR-106b and miR-191) that are capable of distinguishing anaplastic variants from classic and/or desmoplastic MBs. Moreover, the authors found a number of developmentally regulated miRNAs that maintained levels of expression in MB similar to those observed in fetal cerebellum, supporting the notion that MBs exhibit some embryonic features. In MB samples they observed for example impaired expression of several neuronal miRNAs known to be expressed during neuronal development such as (miR-9 and miR-125a, miR-128a, 128b and miR-181b) suggesting that some of these miRNAs might be involved in MB tumorigenesis [91]. Notably all of these miRNAs with altered expressions were identified before in glioblastomas [92], suggesting a common role for these miRNAs in the etiology of malignant brain tumors. The authors selected miR-9 and miR-125a, as down-regulated neuronal candidates, for functional studies. Ectopic over-expression of miR-9 or miR-125a decreased proliferation, augmented apoptosis, and promoted arrest of MB tumor growth suggesting a role for both miRNAs as tumor suppressors [93]. These miRNAs mediated their effects through targeting of the pro-proliferative truncated isoform of the neurotrophin receptor TrkC, which is up-regulated in MB cells. Such findings are in line with those observed in neuroblastomas [91,94]. Consistently, other miRNAs in pediatric brain tumors including MB, such as miR-216, miR-135b, miR-217, miR-592, miR-340 were found to be over-expressed, whereas miR-92b, miR-23a, miR-27a, miR-146b, miR-22) were found to be underexpressed compared to normal brain tissues [95].

In addition to their role in promoting the development of primary tumors, miRNAs have also been implicated in the metastatic dissemination of tumor cells and alterations in miRNA function can influence metastatic potential [96,97]. Among several pro-metastatic miRNAs, miR-21 is up-regulated in a variety of solid tumors, including glioblastoma, breast, lung, colon, prostate, pancreas and stomach cancers [98] and has been causally linked to cell migration of several cancer cell lines [99]. We recently reported the aberrant expression of miR-21 in MB cells and showed that miR-21 regulated the expression of multiple target proteins associated with tumor dissemination, many of which are implicated in MB biology [100]. Importantly, repression of miR-21 decreased MB cell migration *in vitro*, possibly due to diminished repression of the tumor suppressor PDCD4. Thus, miR-21 could function as a pro-metastatic miRNA in MB and therefore raising the possibility that pharmaceutical strategies directed against miR-21 could improve the curability of selected MBs (Table 1 and Table 2).

**Table 1:** Frequently deregulated miRNAs in neuronal embryonal tumors.

Tumor	miRNA regulation	Validated or putative targets	Potential function	References
MB Reviewed in [101,102,103,104]	miR-17/92 Up	TSP-1, Bmi-1, PTEN, PP2A	Enhances proliferation, angiogenesis and confers growth advantage to MBs	[90,105,106]
	Let-7 Down	RAS STAT3	Disregulation leads to a less differentiated cellular state	[107,108]
	miR-199b-5p Down	HES1 Notch pathway ErbB2	Inhibits tumor growth and metastasis. Its restoration blocks expression of several cancer stem-cell genes	[109]
	miR-34a Down	Notch ligand Delta-like 1 (Dll1)	Negatively regulates cell proliferation, and induces apoptosis and neural differentiation in MB cells	[110]
	miR-214 Up	Gli1	Undefined	[27]
	miR-125a Down	t-TrkC	Its restoration decreases proliferation, augments apoptosis, and promotes arrest of MB tumor cell growth.	[107]
	miR-9 Down	REST/NRSF, Hes1	Inhibits MB cell proliferation and promotes differentiation	[107,111]
	miR-125b miR-324-5p miR-326 Down	PKM2, SMO, Notch	Inhibits MB cell proliferation, and reduces the size and number of MB cell colonies	[27,112]
	miR-124a Down	CDK6, SLC16A1, REST, BAF34a, RB1, t-TrkC	Cell cycle regulation. Inhibits proliferation and induces apoptosis	[25,65,88,113,114]
	* (miR-30b and miR-30d) Up	Undefined Both miRNAs are part of an amplicon containing the KHDRBS3 gene on 8q24.22 in MB cell lines	Undefined	[115]
	miR-218 Down	EGFR, Bcl-2, B-catenin and MAPK9	Iinduces apoptosis, inhibits MB tumor growth and invasiveness	[116,117]
	* miR-31 and miR-153 Down	Undefined. however, miR-153 is found in high ErbB2 expressing MB	Undefined	[107,117]
	miR-106b Up	p21	Cell cycle arrest	[107,116]
	miR-128a miR-128b miR-181b Down	Bmi-1	Inhibits growth of MB cells. miR-128a, alters the intracellular redox state and promotes cellular senescence in MB cancer stem cells, which are thought to be resistant to therapy due to their low ROS states	[91,118]
MB Reviewed in [101,102,103,104]	miR-21 Up	PDCD4.	Pro-metastatic miRNA in MB. Involved in mitotic signaling, cell cycle, cell migration	[100]
	miR-183~96~182 cluster Up	Undefined, however knockdown of the full miR-183~96~182 cluster results in enrichment of genes associated with apoptosis and dysregulation of the PI3K/AKT/mTOR signaling axis	Regulates multiple biological programs that converge to support the maintenance and metastatic potential of MB	[119]
	* miR-216, miR-135b, miR-217, miR-592, miR-340 Up	Undefined	Undefined	[95]
	* miR-92b, miR-23a, miR-27a, miR-146b, miR-22 Down	Undefined	Undefined	[95]
	miR-148a. Up.	Undefined	Undefined	[120]
sPNET [103]	* miR-517c miR- 520g Up	Undefined	Undefined	[121]
	* miR-19a, miR-106b and miR-19 Up	Undefined	Undefined	[107]
AT/RT	miR-9 Down	Undefined	Undefined	[107,111]
	miR-517c, miR-520g Up	miR-517c WNT/JNK signaling miR-520g ABCG2.	Undefined	[121,122]
	miR-221, miR-222 Up	p27^Kip1^	Promotes proliferation through cell cycle progression in AT/RT cells	[123]
	* miR-520b, miR-629, miR-498, miR-373 Up	Undefined	Undefined	[95]
	* miR-140, let-7b, miR-139, miR-153, miR-376b Down	Undefined	Undefined	[95]
	let-7 miRNA family Down	HMGA2	Suppresses proliferation and colony formation and abolishes the invasive potential	[124]
NB Adapted from [102]	miR-17-92 Up	DKK3, CDKN1A, BIM, ER-α, MEF2D	Increases proliferation, decreases apoptosis and inhibits TGF-β signaling. miR-17-5p inhibition abolish growth of therapy-resistant NB *in vitro* as well as *in vivo*	[125,126,127,128,129,130,131]
	miR-124 Undefined	AHR	Inhibits apoptosis and differentiation	[127,128,129,130]
	miR-181 Up	ATM	Undefined	[125]
	miR-21 Up	PTEN	Promotes proliferation, and decreases sensitivity to chemotherapy	[127,128,129,130]
	miR-380-5p Up	P53	Decreases apoptosis	[132]
	miR-15a Up	RECK	Promotes migration through the induction of MMP-9 expression	[133]
	* miRNA-125a/b Induced upon treatment with RA	Modulate expression of the TrkC neurotrophin receptor	Undefined	[94,134]
	miR-184 Undefined	Akt2 downstream effector of the pro-survival pathway PI3K	Pro-apoptotic effects. Mediates proliferation reduction and inhibition of tumor growth in an orthotopic murine model of NB	[135]
	miR-128 Undefined	Reelin and DCX	Mediates differentiation, reduces motility, invasiveness, and growth of NB cells	[136]
	miR-34a miR-34c miR-410,487b Down	E2F3, BCL2, CCND1, CDK4, MYCN, P53	Inhibits proliferation and induces apoptosisEctopic expression of miR-34a leads to cell-cycle arrest in NB lines	[127,134,135,137,138,139]
	miR-542-5p Expressed in favorable NB, absent in unfavorable NB	GRIN3A, SH3GLB2, SNIP	Inhibits tumor growth, invasiveness and metastasis.	[125,140]
	miR-628 Expressed in favorable NB, absent in unfavorable NB	Undefined	Undefined	[125,140]
	miR-let-7 Down-regulated in NB with MYCN amplification	MYCN	Inhibits proliferation and induces differentiation. Altered expression of let-7 leads to a less differentiated cellular state	[141]
	miR-101 Down-regulated in NB with MYCN amplification	MYCN	Inhibits proliferation and clonogenic growth	[141]
NB Adapted from [102]	miR-885-5p Down	CDK2, MCM5	Inhibits cell cycle progression and survival	[142]
	miR-27b Down	PPARγ	Inhibits tumor growth	[143]
	miR-138 Down	Undefined	Decreases viability and growth	[144]
	miR-137 Down	KDM1A	Inhibits proliferation and induces apoptosis	[145]
	miR-204 Down	BCL2, NTRK2.	Increases sensitivity to cisplatin and etoposide	[146,147]
	miR-190 Down in aggressive NB with unknown mechanism	NEUROD1	Inhibits TrkB pathwayand aggressive phenotypes.	[147]
	miR-10a/b Down	NCOR2 miR-10a/b	Induces differentiation	[148]
	miR-335 Down	ROCK1, MAPK1, LRG1	Inhibits migration and invasion	[149,150]
	miR-363 Down	Undefined	Inhibits invasiveness and metastasis	[149]
	miR-9 Down	MMP-14	Inhibits the invasion, metastasis, and angiogenesis	[151]
	miR-145 Down	HIF-2α	Inhibits NB cell growth, invasion, metastasis and angiogenesis	[152]
	* miR-140 let-7b miR-139 miR-153 miR-376b Down	Undefined	Undefined	[95]
	let-7a3/let-7b miRNA Down	HMGA2	Undefined	[124]

* Poorly characterized miRNA/s in the specific tumor setting.

**Table 2:** Clinical relevance of miRNAs in NB [102,125,130,131,153,154,155,156,157,158,159].

miRNAs up/down regulated in high-risk NB	miRNAs up-regulated in metastatic NB	miRNAs up/down regulated as a marker for poor survival of NB	miRNAs up/down regulated in unfavorable NB
↑miR-18b, ↑miR-20a, ↑miR-22,	miR-24,	↑miR-380-5p, ↑miR-17-92	↑miR-181, ↑miR-21,
↑miR-92a, ↑miR-181a, ↑miR-181a-2,	miR-92b,	cluster, including ↑miR-17-5p,	↓miR-542-5p,
↑miR-203, ↑miR-373, ↑miR-383,	miR-99b,	↑miR-18a, ↑miR-19a,	↓miR-628,
↑miR-422a, ↑miR-876-5p,	miR-129-3p,	↑miR-20a and ↑miR-92 and	↓miR-410,
↑miR-1208, ↑miR-1285, ↑miR-1290,	miR-130b,	↓miR-542-5p, ↓miR-497	↓miR-487b,
and ↑miR-129 and ↓miR-30b,	miR-342-3p,	miR-18a and miR-19a	↓miR-30c, ↓miR-149,
↓miR-146a, ↓miR-190, ↓miR-204, ↓miR-215, ↓miR-299-5p, ↓miR-362, ↓miR-382, ↓miR-411, ↓miR-424, ↓miR-425, ↓miR-487b, ↓miR-532, ↓miR-629, ↓miR-656, ↓miR-660, ↓miR-668, ↓miR-744, ↓miR-758, ↓miR-873, ↓miR-885-5p, and ↓miR-1197, miR-30c, ↓miR-149, ↓miR-195, miR-324-5p and ↓miR-331	miR-345, miR-483-3p, and miR-486-5p. miR-17-92	(correlates with increased event-free survival and favorable disease outcome). ↑miR-184 (increases overall survival). ↑miR-204 (associated with a favourable clinical outcome and increases sensitivity to chemotherapy).	↓miR-195, ↓miR-324-5p ↓and miR-331

↑: Upregulated, ↓: Downregulated.

#### 5.1.2. MB Subgroups Have Distinct miRNA Profiles

Genome-wide miRNA expression studies have revealed close associations between miRNA clusters and MB tumor subtypes as well as clinical subgroups [90,107,119,160]. Additionally, miRNA profiling studies demonstrated that it is possible by miRNA signatures to discriminate MB histological subtypes. While miR-21, a known oncomir [161], was up-regulated across all MB subgroups relative to normal cerebellum, Northcott and colleagues reported that miR-17/92 cluster was found to be significantly up-regulated in SHH-driven MB [90,162]. The group concluded that miR-17/92 is a positive effector of SHH-mediated proliferation and that aberrant expression/amplification of this miRNA confers a growth advantage in a specific subpopulation of MBs. Their results were confirmed by the discovery that miR-17/92 promoted proliferation of MB cell lines *in vitro* and contributed to the development of MB *in vivo* [90,162]. In other studies microRNAs encoded by the miR-17/92 and miR-106b-25 clusters were found to be over-expressed specifically in human and mouse SHH MBs [107,163]. Consistent with its high expression in SHH MB, enforced expression of the miR-17/92 cluster, together with mutation of Ptch1+/−, accelerates the onset and penetrance of SHH MB [162]. Tumor progression can be inhibited by using miRNA inhibitors that target the seed sequence of miR-17/20 and miR-19a/b in SHH MB cells *in vitro* and in animals bearing flank or cortical SHH MB allografts [164]. These data suggest that miRNAs inhibitors targeting the miR-17/92 cluster family could be therapeutically useful in SHH MB and other cancers that over-express members of this cluster family [165]. Weeraratne and colleagues have reported that increased expression of the miR-183~96~182 cluster characterizes non-SHH MBs. This cluster of miRNAs was found to be associated with MB subgroups characterized by genetic amplification of MYC [119]. Remarkably, miR-182 is known to promote metastasis, a hallmark of aggressive MBs [166]. Three other microRNAs were found to be differentially regulated in MYC*-*over-expressing MBs compared to MBs in which MYC is not over-expressed [107]. However, whether these microRNAs are directly regulated by MYC is yet unknown, reviewed in [165]. In another publication Cho *et al.* discovered a previously unidentified molecular subgroup, characterized genetically by MYC copy number gains and transcriptionally by enrichment of photoreceptor pathways and increased miR-183-96-182 expression. This newly discovered subgroup is associated with significantly lower rates of event-free and overall survivals [167]. For the WNT MB subgroup, Gokhale *et al.* reported a distinctive microRNA signature associated with the WNT signaling pathway. In their study miR-193a, miR-224/miR-452 cluster, miR-182/miR-183/miR-96 cluster, and miR-148a having potential tumor/metastasis suppressive activity were found to be over-expressed in the WNT signaling associated MB [120].

In summary, the altered miRNA expression signatures in distinct MB subtypes highlight the functional significance of these novel oncogenic molecules in MB tumor formation and progression. Such discoveries not only provide new insights into the molecular pathogenesis of MB tumors, but also raise hopes for translating the increasing knowledge of miRNA expression and functions into diagnosis, prognostication and therapy.

### 5.2. Supratentorial Primitive Neuroectodermal Tumors

The supratentorial primitive neuroectodermal tumors (sPNET), which represent 3%–7% of all pediatric brain tumors, are a group of highly malignant lesions primarily affecting young children. Although these tumors are histologically indistinguishable from infratentorial MB, they often respond poorly to MB-specific therapy [168]. Indeed, the very few existing molecular genetic studies indicate that sPNET have cytogenetic profiles that are different from those of MBs, thus pointing to unique biological derivation for the supratentorial PNET. sPNET are a heterogeneous group characterized by primitive neuroepithelial cells with variable neuronal, glial, or ependymal differentiation [121]. Unlike AT/RT, which are defined by INI1 gene alterations [169], sPNET may pose significant diagnostic challenges due to lack of characteristic genetic or immunohistochemical markers [121]. Due to the shortage of these tumors, comprehensive genetic studies of a substantial number of sPNETs have not yet been undertaken. Therefore, the lack of insight into the molecular pathogenesis of sPNET has become a major obstacle toward development of disease-specific models and treatments.

#### miRNAs Aberrantly Expressed in sPNET

Owing to the rarity of these tumors and disagreement about their histopathological diagnoses, very little is known about the molecular characteristics of sPNET. Additionally, the role of miRNAs in sPNET was not yet established until recently [170]. The chromosome 19 microRNA cluster (C19MC) is the largest human microRNA gene cluster described so far. This miRNA cluster spans 100 kb at human chromosome 19q13.42 and comprises 54 tandemly repeated miRNA genes [171]. Amplification of a miRNA cluster at 19q13.42 has been identified recently as a genetic hallmark of sPNET, and it is considered a unifying molecular diagnostic marker for these tumors [121,171,172]. Very recently Li *et al.* established that amplification of C19MC was present in aggressive sPNET tumor cells [121]. They showed that miR-517c and/or 520 g in the C19MC group could stimulate growth and prevent development of normal human neural stem cells into more mature types of brain cells. They also reported that constitutive expression of miR-517c or 520 g promotes *in vitro* and *in vivo* oncogenicity. Furthermore, the group showed that C19MC miRNA oncogenes may confer aggressive tumor phenotype by altering signaling pathways that regulate normal neural stem cell growth. Their data describe for the first time miR-517c and 520 g as oncogenes in sPNET and highlight C19MC as an attractive candidate biomarker and therapeutic target for aggressive pediatric brain tumors. Interestingly, Kleinman and colleagues reported recently that embryonal tumors with multilayered rosettes (ETMR), an aggressive pediatric brain tumors of sPNET variant that originate from early neural stem cell precursors, are characterized by a recurrent fusion that joins the promoter of a gene with brain-specific expression (TTYH1) to C19MC. This fusion event, which is unique to ETMRs, drives high levels of expression of the miRNA cluster in these tumors, reawakening a transcriptional program that is normally only active during the early stages of embryonic development [173,174]. Together these findings support the emerging theme that miRNAs are important determinants of sPNET embryonal tumor biology.

### 5.3. Atypical Teratoid/Rhabdoid Tumors (AT/RT)

AT/RT are rare but highly aggressive tumors that typically occur in infancy or early childhood [175,176]). Rhabdoid tumors predominantly arise in the kidney and brain, but they can also be found in a deep axial location, such as the neck or the paraspinal region [177]. Different forms of rhabdoid tumors can be similar in their aggressiveness, histological features, and loss of function of *INI1/hSNF5* mapping on chromosome 22 [178,179]. From the clinical experience, infants and children with rhabdoid tumors respond very poorly to chemotherapy and radiotherapy [180,181,182], although this remarkable resistance to both cytostatic drugs and radiotherapy has not yet found convincing explanation at the molecular level.

#### miRNAs Aberrantly Expressed in AT/RT

To find new potential therapeutic targets for the treatment of AT/RT, Sredni and colleagues [123] investigated differentially expressed miRNAs when comparing four AT/RT with six MB and normal brain samples. Within the top differentially expressed miRNAs, the deregulated expression of miR221/222 was demonstrated to inhibit the expression of the tumor suppressor and inhibitor of cell cycle p27Kip1. These results suggest that miR-221/222 over-expression might be one of the factors contributing to oncogenesis and progression of AT/RT through p27Kip1 down-regulation. The authors suggested that anti-miR221/222 therapy might be an option for the treatment of these very aggressive and unresponsive tumors. In a comprehensive study Birks *et al.* [95] compared miRNAs expression in pediatric brain tumors and normal tissue controls by microarray. Their result showed that miR-129, miR-142-5p, and miR-25 were differentially expressed in every pediatric brain tumor type compared to normal tissue controls. In AT/RT miR-520b, miR-629, miR-221, miR-498, miR-373* were up-regulated while miR-140, let-7b, miR-139, miR-153, miR-376b were under-expressed. Zhang *et al.* [124] have recently searched for novel genomic aberrations by investigating the copy number and expression alterations of let-7a3/let-7b miRNA and correlated these with expression of high-mobility group AT-hook 2 (HMGA2) oncoprotein, a target of let-7 miRNA family, in 18 AT/RT samples. Their analysis demonstrated that HMGA2 was highly over-expressed in 83.3% (15/18) of AT/RT tissues while let-7a3/let-7b miRNA copy number and expression were reduced. Restoration of let-7 miRNA or knockdown of HMGA2 expression significantly suppressed proliferation and colony formation and almost abolished the invasive potential of G401 Wilms’ tumor cell line. The authors suggested that HMGA2 oncoprotein plays critical roles in the pathogenesis of AT/RT development and reconstitution of let-7 miRNA may provide a novel therapeutic strategy for the treatment of AT/RT patients.

### 5.4. Neuroblastoma

NB is the most common extra-cranial solid tumor in childhood, accounting for approximately 7%–10% of pediatric cancers and 15% of all cancer-related deaths in patients less than 15 years old [183,184,185]. NB is an extremely heterogeneous disease both biologically and clinically [183,184,186]. NB is thought to be an embryonal tumor that is derived from precursor cells of the peripheral (sympathetic) nervous system [2,187]. The tumor can arise anywhere along the sympathetic chain but is most frequently in the adrenal medulla and paraspinal ganglia [187,188] reviewed in [189]. The clinical outcome of NB can range from complete regression (mainly in infants) to rapid tumor progression and metastasis with poor prognosis [190]. Many genetic abnormalities have been identified in NB tumors, including amplification of the MYCN proto-oncogene (25%–33% of patients) and consistent areas of chromosomal deletion and rearrangement that result in loss of 1p36 (25%–35%), 11q23 (35%–45%), and 14q23 (16%–27%), as well as unbalanced gain of 17q22 (~50%). Many of these abnormalities are powerful prognostic markers and are highly related to clinical outcome. For example, NB tumors which harbor 1p36 LOH and MYCN amplification are usually advanced-stage aggressive tumors that are frequently metastatic and generally respond poorly to chemotherapy/irradiation [183,184]. Amplified *MYCN* has been proposed as an example of the first clinical application of cellular oncogenes, and is now established as a powerful molecular parameter to determine patient prognosis, as well as a basis for patient stratification [183,191,192].

#### MicroRNAs Aberrantly Expressed in NB

Aberrant regulation of miRNA expression has been implicated in the pathogenesis of NB (Table 1 and Table 3), and miRNA expression profiles have been correlated with prognosis, and cell differentiation, and apoptosis [193], suggesting that miRNAs could function as tumor suppressors or oncogenes in NB. Guo *et al.* compared miRNA expression patterns of primary and metastatic NB tumors and found significant changes of 54 miRNAs [153]. MiR-10b, miR-29a/b, miR-335, which are known to promote metastasis were up-regulated in metastatic NB tumors. In contrast, miR-7, miR-338-3p and the let-7 family were three of the top 10 down-regulated miRNAs in the metastatic group. Interestingly, these miRNAs have been shown to play anti-metastatic roles in other tumors [194], and target gene analysis of these miRNAs revealed that many of these targets are related to metastasis. For instance, both caspase-8 and integrin beta1 are predicted targets of miR-29a and miR-29b, miRNAs known to promote metastasis in breast cancer [195] reviewed in [189]. While the MYCN protein is established as an important regulator of several miRNAs involved in NB tumorigenesis, tumor suppressor miRNAs have also been documented to repress MYCN expression and inhibit cell proliferation of MYCN-amplified NB cells [196]. Several studies published during the last 5 years have implicated MYCN in the regulation of the expression of several miRNAs, many of which have important roles in cancer progression. miRNA induction by MYCN is associated with a widespread repression of coding genes and disease-relevant pathways in NB cells, suggesting that miRNA-controlled regulation of certain groups of miRNAs may function as an additional mechanism of MYCN-induced oncogenicity. Indeed, among the targets of MYCN-responsive miRNAs (mir-17/92 cluster, mir-9 and mir-421) there are tumor suppressors and genes involved in the metastatic process. Fontana and co-authors [197] and Schulte and colleagues [198], showed that miRNAs of the miR-17/92 cluster are over-expressed in NB tumors and cell lines with high MYCN expression. Other studies have confirmed direct binding of MYCN to the miR-17-92 promoter [154,199], as well as a positive correlation between expression of MYCN and members of the miR-17/92 cluster in NB primary tumors and/or cell lines [125,126,154,200,201,202]. On the other hand, miRNAs with tumor suppressor functions (mir-184 and mir-542-5p) have been shown to be inversely correlated to MYCN expression [140,155,193,203]. In fact, miRNA repression seems to be the predominant consequence of MYCN activation, as high MYCN expression is associated with a global repression of miRNAs. Vice versa, miR-34a, miR-101 and let-7 have been documented to target MYCN mRNA, resulting in reduced MYCN expression and decreased proliferation of MYCN-amplified NB cells [196].

**Table 3:** Biological and clinical relevance of miRNAs in MB.

miRNAs associated withsignaling pathways involved in MB classification/pathogenesis	miRNAs associated with MB and neuronal stem cell biology		miRNAs associated with metastasis in MB		miRNAs associated with risk stratification/outcome prediction
↓miR-125b, ↓miR-326, and ↓miR-324–5p, ↑miR-214 and ↓miR-92 (associates with SHH) [27]	miR-17/92 (promotes neural stem cells development, modulating its cell-fate decision and are also involved in cancer stem cells and in MBs biology) [204]		miR-21, miR-182 (promotes metastasis)miR-182 contributes to leptomeningeal metastatic dissemination in non-SHH-MB [166]		Tumor suppressor miR-31 and miR-153 (down-regulated in clinical high risk MB patients) [91,107]
↑miR-183~96~182 (associates with genetic amplification of MYC in the most clinically aggressive MB subgroup) [119,166]	miR-199b-5p (negatively regulates MB tumor stem-cell positive to CD133 antigen) [109]		miR-193a, miR-224/miR-452 cluster,, and miR-148a (potential metastasis suppressive activity) [160]		Oncogene miR-183~96~182 (highly expressed in the most clinically aggressive MB subgroup) [119]
↑miR-17-92 (correlates with SHH as well as c-MYC/MYCN activation in MB) [162]	miR-125b, miR-324-5p, and miR-326 (regulate SHH signaling in cerebellar granule neuron precursors and MB cells) [27]		miR-199-5p (confreres inhibition of MB metastasis) [109]		Tumor suppressor miR-9 (downregulation is a predictive marker for poor prognosis in MB) [111]
miR-10b, ↑miR-135a, miR-135b, ↑miR-125b and ↓miR-153 (associated with ErbB2 overexpressing MB tumors) [91,107]	miR-7, miR-9, and ↓miR-124 (associated with MB and Neuronal Differentiation) [103,104,111]				Tumor suppressor miR-199-5p (low expression is a predictor for a poor-risk MB class) [109]
↓miR-181b, miR-128a, and ↓miR-128b (associated with c-MYC overexpressing MBs) [91,107]					miR324-5p and miR326 (promote progressive events in MBs) [27]
↓miR-199b and ↓miR-326 miR34a (associated with Notch Signaling pathway) [109]					Tumor suppressor miR-128b miR-181b low expression (correlates with MB disease risk) [91,107]
↑miR-193a, ↑miR-224/↑miR-23b, ↑miR-365 and ↑miR-148a (over-expressed in the WNT signaling associated MB) [120,160]					
↑miR-let7g, ↑miR-19a, ↑miR-106b and ↑miR-191distinguish MB differing in histotypes (anaplastic, classic and desmoplastic [107]					

↑: Upregulated, ↓: Downregulated.

## 6. Role of miRNAs as Biomarkers

With the increasing implication of miRNAs in cancer development and progression, significant efforts are underway to use miRNAs as novel biomarkers with clinical applications [205,206]. miRNA expression is dynamic: many miRNAs are deregulated in early stages of tumor development and up-regulated during cancer progression and could thus be of potential diagnostic utility [207,208]. miRNA expression profiling has been used to characterize embryonal and differentiated tissues of the nervous system [56], to discriminate cancer from normal tissue [97,161] and it can also be used to differentiate primary from metastatic brain tumors [209]. Moreover, miRNAs may be used as biomarkers to identify individuals with increased disease risk, as well as novel prognostic factors [210]. Of note miRNAs can also be detected circulating in several body fluids, including plasma, serum, cerebrospinal fluid, urine and saliva [211,212,213,214], reviewed in [215,216]. There are significant differences between the circulating miRNA expression profiles of healthy individuals and those of patients. Consequently, circulating miRNAs are likely to become a novel class of non-invasive and sensitive biomarkers [217]. To be classified as an ideal biomarker, it is crucial that miRNAs show satisfactory predictability and the ability to be inspected during onset, progression and/or regression of the disease. The ease of obtaining and detecting such biomarker from clinical samples is also important. In that regard, circulating miRNAs are particularly promising potential biomarkers in diagnosing diseases because they exhibit an unexpected stability in various body fluids [206,218]. Importantly, obtaining clinical samples that contain circulating miRNAs is a non-invasive and simple process. Moreover, the miRNAs can then easily be detected using quantitative reverse transcription-polymerase chain reaction (qRT-PCR), an efficient and easy to apply technique. These advantages indicate that circulating miRNAs have the potential to be useful candidates for diagnosis and other clinical applications in human diseases. However, because of the small amount of circulating miRNAs, extracting miRNA from body fluids remains technically challenging and needs to be improved. Despite the limitations, the application value of circulating miRNAs in various diseases is gradually being uncovered, reviewed in [217]. In fact the use of circulating microRNAs as potential biomarkers is a rapidly evolving field of study [206,219] and the number of recent publications has increased rapidly over the past two years. In the context of CNS diseases, several studies have demonstrated significant presence of certain miRNAs in CSF samples from patients with CNS lymphoma, glioma [214], metastatic brain cancers [213] and Alzheimer’s disease [220]*.* However, the true source of miRNAs in the body fluids and their exact secretory mechanism is relatively unknown [221]. A number of hypotheses have been put forward, such as that circulating miRNAs are present as a result of cell death and lysis or as a result of being actively secreted by cells [222]. The last hypothesis involves either encapsulation of miRNAs into microvesicles such as exosomes [223,224] or alternatively secreted as microvesicle-free (naked) miRNAs [225]. Tumor-derived exosomes containing miRNAs are generally considered pro-tumorigenic. Recent studies have shown that miRNAs contained in tumor-derived exosomes are able to transfer their oncogenic activity to recipient target cells and promote cancer stimulatory activities, such as proliferation, extracellular matrix remodeling, migration, invasion and angiogenesis, and contribute to the pre-metastatic niche formation for the promotion of metastasis, reviewed in [226]. This potential exchange of miRNAs is an exciting and novel dimension to the regulation of a cell’s phenotype [227] and is particularly important in cancers that have a propensity for dissemination such as embryonal tumors.

## 7. Risk Stratification and Outcome Prediction

### 7.1. Prediction of Clinical Outcome in MB

Risk stratification and prognosis assessment have become a major concern in the era of personalised medicine. Gene expression profiling has reached a plateau in this regard, although recent miRNA studies show great promise [90,119,160] reviewed in [21]. Ferretti *et al.* identified specific miRNA expression patterns, which distinguish MB differing in histotypes (anaplastic, classic and desmoplastic) and in disease-risk stratification. The authors reported four miRNAs (miR-let7g, miR-19a, miR-106b and miR-191) that are significantly up-regulated in anaplastic *versus* desmoplastic MBs, miR-let7g and miR-106b were differentially expressed between desmoplastic and classic tumors while only miR-19a was up-regulated in anaplastic *versus* classic MBs [107]. Importantly their analysis identified both miR-31 and miR-153 as down-regulated in clinical high risk MB patients [91,101,107]. In another study Weeraratne and colleagues reported that increased expression of the miR-183~96~182 cluster of miRNAs has been noted in the most clinically aggressive subgroup and associated with genetic amplification of MYC [119]. Our group recently identified miR-9 as a methylation-silenced tumor suppressor that could be a potential candidate predictive marker for poor prognosis of MB [111]. The investigation demonstrated that LC/A MB samples possess lower miR-9 expression compared to the other variants whereas miR-9 under-expression was associated with poor prognosis in 34 patient samples of MB. The lower overall survival probability of patients with low miR-9 expression that also tends to have a more severe pathological grade suggests a strong trend towards prognostic significance. Moreover, in line with previous reports high expression of the main miR-9 target gene (HES1) correlated significantly with lower overall survival in a distinct cohort of 129 MB samples [167]. Together these data indicate that low expression of miR-9 and the consequently increased expression of its target gene HES1 could be associated with worse MB clinical outcome. In addition, Garzia *et al.* reported that the expression of miR-199-5p (that also targets HES1) is a robust predictor for a poor-risk class and correlates with metastatic spread in patients with MB [109]. In summary, although further verification is required to validate the role of miRNAs in MB risk stratification and outcome prediction, the above mentioned data imply that in the very near future miRNAs may have a wide clinical applicability as potential diagnostic and prognostic biomarkers for MB patients.

### 7.2. Prediction of Clinical Outcome in NB

Several miRNAs have been found to have links with the outcome of NB patients [134,228]. In searching for miRNA target signatures in advanced stage NBs, Guo *et al.* show that 54 different miRNAs are significantly altered in metastatic as opposed to primary NB tumors [153]. In another study by Pray and colleagues, miR-542-5p over-expression decreases the invasive potential of NB cells [140]. The authors demonstrated that lower expression of miR-542-5p is highly associated with poor patient survival, while ectopic over-expression of this miRNA decreases the invasive potential of NB cell lines *in vitro*, along with primary tumor growth and metastases in an orthotopic mouse xenograft model. In a parallel study Schulte *et al.* confirmed that miR-542-5p also discriminated between local and metastatic disease and was inversely correlated with MYCN amplification and event-free survival [155]. Chen *et al.* identified several miRNAs differentially expressed in different prognostic subtypes of NB. Usually, most of them are down-regulated, as result of MYCN amplification in NB high-risk subgroup. Combined with MYCN amplification, miRNAs down-regulation predicts a poor prognosis in NB patients [193]. In agreement with this trend Creevey *et al.* determined that miR-497 expression was significantly lower in high-risk MYCN amplified tumors and that low miR-497 expression was associated with worse event-free survival and overall survival in their cohort [229]. The group showed that miR-497 restoration in MYCN amplified NB cells increases NB apoptotic cell death *in vitro*. Another study showed high expression of MRHG1 was significantly associated with high-risk groups of NB, which was caused by dysregulation of miR-17, miR-18a, miR-19a, miR-20a and miR-92a [117]. The oncogenic activity of miR-18a and miR-19a was proven by Lovén *et al.* miR-18a and miR-19a have been found to target and repress the expression of estrogen receptor-alpha (ESR1) and high ESR1 expression correlates with increased event-free survival in NB patients and favorable disease outcome [154]. In preclinical studies Tivnan *et al.* showed that miR-184 significantly reduces tumor growth and increases overall survival in an orthotopic murine model of NB [135]. Others demonstrated that miR-204 increases sensitivity of NB cells to cisplatin and is associated with a favourable clinical outcome [146]. Deep sequencing by Schulte *et al.* revealed differential expression of miRNAs in favorable *versus* unfavorable NB. Oncogenic miRNAs of the miR17/92 cluster and the miR-181 family were found to be over-expressed in unfavorable NBs. In contrast, the putative tumor suppressive miR-542-5p and miR-628 were expressed in favorable NBs and virtually absent in unfavorable NBs [125]. Predicting outcome of individual NB patients within risk groups remains challenging and established cytogenetic abnormalities such as MYCN amplification or segmental chromosomal abnormalities do not identify all patients with adverse outcome. Deregulation of miRNAs is associated with the development and progression of NB, hence in the next decade miRNAs are expected to have a significant effect on clinical oncology for the diagnosis and prognosis of NB, and may serve as novel targets for the treatment of high-risk NB patients.

## 8. Pros and Cons of the miRNAs as Biomarkers for Embryonal Neural Tumors

If the ultimate expectation of miRNAs as biomarkers is to enhance the ability to manage the patient optimally, a key question to ask is whether miRNA biomarkers would significantly improve the diagnostic workup and eventually lead to positive targeted clinical outcome when compared to already in-use diagnostic standards.

Four major risk stratification systems for NB patients are currently being used by combining various clinical, histopathological and molecular markers, such as established cytogenetic abnormalities, mainly chromosomal abnormalities and MYCN amplification. Accordingly, different therapeutic schemes exist ranging from wait-and-see approaches to intensive multimodal therapies. Although the currently used risk stratification systems are considered useful, patients receiving the same treatment can have markedly different clinical courses [230], and established cytogenetic abnormalities do not identify all patients with adverse outcome. On the contrary, molecular-based MB risk stratification is not yet in use in the clinics. Nevertheless a current consensus identifies the existence of four major subgroups of MB, with good prognosis for those with WNT tumors, intermediate prognosis for those with SHH and Group 4 tumors and poor prognosis for those with Group 3 tumors [87]. However, evidence suggests clinical heterogeneity within these core subgroups [86,167,231] and importantly the putative markers are still waiting to be validated in prospective clinical trials.

Considering the limitations of current cancer conventional biomarkers, the use of miRNAs as tumor markers for diagnosis and prognosis has aroused intense research interests in particular after the outstanding advances in miRNA technologies, such as miRNA microarrays, specific quantitative PCR of miRNA, bead-based miRNA profiling, and antisense technologies. Advanced technologies not only help to identify the oncogenic and tumor suppressor potentials of miRNAs, but also assist in providing a more comprehensive understanding of their underlying mechanisms and pathways. miRNA microarrays can identify the expression of several hundreds miRNAs genes in the same sample at once while requiring only small amounts of total RNA. The widespread and comprehensive use of these high-throughput arrays has enabled the quantification of miRNA expression and the identification of miRNAs uniquely expressed within specific disease [205]. Moreover, profiling of miRNA expression patterns was shown to be more useful than the equivalent mRNA profiles [232]. As such, miRNA expression “signatures” are expected to offer serious potential markers for diagnosing and prognosing cancers of any provenance. However this does not go without restrictions. From clinical sensitivity and specificity point of view although it is suggested that miRNAs would provide increased sensitivity because miRNA profiles have been demonstrated to be pathognomonic, or tissue-specific; however, this does not necessarily translate into disease specificity [233]. The other restrictions that hinder the use of miRNAs as biomarkers include those inherently associated with conventional biomarkers. For example detection and quantification of an ideal marker should be simple, accurate and reproducible. However, miRNA detection currently falls short of most of these requisites. Biological variance, which has been largely neglected, represents an extremely important variable that affect the clinical utility of miRNAs as reliable markers. Currently there are no universally implemented guidelines for specimen collection, preparation and preservation. By using material from different sources obtained with dissimilar techniques, samples are more likely to show wide variability in profile. These are especially pronounced when a highly sensitive technique like sequencing is used. Moreover, differences in specimen type can also introduce a profound effect on miRNA concentrations as well as expression levels. In addition to this pre-analytical variability, there are also variables specifically associated with miRNAs extraction and quantification methods. Often there is a low correlation of results obtained from different platforms, and sometimes even within the same platform, using reagents from different vendors. Data normalization, an often underestimated aspect of data processing, is another crucially important concern in obtaining accurate results. Hence baseline parameters such as pre-analytical, intra-individual, and inter-individual variability of miRNAs must be explored and defined. Steps must be taken to carefully select miRNA-profiling platforms and data analysis methods for the acquisition of clinically meaningful and dependable data. Furthermore, standardization of miRNAs extraction assays, quantification methods as well as miRNA biomarker evaluation is definitely needed. Finally and importantly, to date, most of the investigations into the use of miRNAs as biomarkers have been performed mainly in cell lines or animal models while other studies included only small cohorts of patient samples that were not validated in prospective clinical trials. A final decision for human use must be based on results derived from clinical trials with an adequate number of patient samples that represent a proof of concept for the field of miRNA as biomarkers.

## 9. miRNAs as Potential Cancer Therapeutics

The rationale for using miRNAs as anticancer drugs is based on the findings that miRNA expression is deregulated in cancer and that cancer phenotype can be changed by targeting miRNA expression. The evidenced deregulation of miRNAs in embryonal tumors [103,104] (Table 1) together with their association with the risk stratification and outcome prediction in these deadly cancers of childhood [103,104] (Table 2 and Table 3), recommends miRNAs for targeting as novel therapeutic approaches and as effective treatment tools. Depending on expression levels and function of miRNAs, two approaches to develop miRNA-based therapies can be envisaged: antagonists (inhibition of oncogenic miRNAs) and mimetics (replacement of tumor suppressive miRNAs) [234]. Table 4 illustrates preclinical analysis of diverse aberrantly expressed miRNAs in MB and NB that their therapeutic effects, which were determined with the tactics of reversing the expression level of these miRNAs *in vitro* and/or *in vivo*, have proven to be promising, reviewed in [102,235]. 

**Table 4:** Example of preclinical functional studies of miRNAs in MB and NB [235].

microRNAs	Theraputic Potential	Models Investigated	Reference
* miR124	The adverse prognostic markers in MB (CDK6 SLC16A1D) are regulated by miR-124 Overexpression of the tumor suppressor miR124 decreases MB cell growth *in vitro* and in xenograft tumors in mice suggesting possible use of miR-124 restoration in MB therapy	D283, D341, D384, D425, D458, ONS-76 MB cell lines. D425 xenograft tumors in mice.	[88,113]
* miR199b-5p	miR199b-5p target Notch effector HES1, which is involved in MB pathogenesis. miR199b-5p overexpression impairs the clonogenic potential of MB cell lines. Moreover MB tumor infection by adenovirus carrying miR199b-5p in a xenograft model reduces the tumor burden *in vivo* suggesting possible use of miR199b-5p as an adjuvant therapy	Daoy cell line and Daoy xanograft mouse model.	[109]
* miR34a	miR-34a is a regulator of the Notch ligand Dll1. Down-regulation of Dll1 expression by miR-34a negatively regulates cell proliferation, and induces apoptosis and neural differentiation in MB cells. In addition infection of adenoviruses carrying the precursor miR-34a induces neurogenesis, reduces tumor burden, and confers chemosensitivity in mice xenografts,	Daoy cell line and Daoy xanograft mouse model.	[236]
* miR125b, miR324-5p, miR326	miR125b, miR324-5p, miR326 suppress Hedgehog signaling which controls cerebellar granule cell progenitor (GCP) development as well as neoplastic transformation into MB. Overexpression of these miRNA increases GCP differentiation and inhibits MB cell proliferation, and colonies formation.	Daoy and D283 cell lines.	[27]
* miR128a	By targeting the Bmi-1 oncogene, miR-128a inhibits growth of MB cells and alters the intracellular redox state of the tumor cells and thereby promotes cellular senescence in MB cells.	Daoy, ONS-76 cell lines.	[118]
* miR383	Tumor suppressor miR-383 is down-regulated in MB. Overexpression of miR383 inhibits MB cell growth through targeting PRDX3	Daoy, D283, D341, D384, D425, D458, ONS-76 cell lines.	[237]
* miR9, miR125a	miR-9 and miR-125a are tumor suppressors that are downregulated in MB patient with poor survival. Rescued expression miR-9 and miR-125a promotes MB cell growth arrest and apoptosis by targeting truncated TrkC isoform or notch signal via His1.	D283 and Daoy cell lines. D341, D425	[107,111]
* miR218	miR-218 is significantly underexpressed in MB. miR-218 target multiple cancer associated genes such as REST, CDK6, RICTOR and CTSB in MB cells. Re-expression of miR-218 resultes in a decrease in MB cell growth, colony formation, cell migration, invasion, and tumor sphere size.	Daoy, D283, D341, D425, D458 and UW228 cell lines.	[238]
** miR-17-92	Activation of miR-17-92 cluster is known as a marker for poor prognosis and poor survival of NB. miR-17-92 affect multiple cancer pathways including DKK3, CDKN1A, BIM, ER-α, MEF2D Its activation increases proliferation, decreases apoptosis and inhibits TGF-β signaling. Aggressive NB evade the cytostatic TGFβ-pathway through miR-17-92 directed targeting of the pathway. Reactivation of TGFβ-signaling through miR-17-92 inhibition could be a promising therapeutic approach for NB	SHEP NB cell line xanograft mouse model.	[131]
** miR-21	Oncogenic miR-21 promotes the proliferation and decreased sensitivity to chemotherapy of human NB cells. Ectopic expression of pre-miR-*21* lower the level of tumor suppressor PTEN mRNA and protein and in NB cells. Transfection of anti-miR-*21* increases the PTEN expression. However, others reported that inhibition of miR-21 did not affect proliferation of NB cells, suggesting that the precise biological functions of miR-21 in NB still warrant further studies.	SH-SY5Y and BE(2)-M17 NB cell lines	[129]
** miR-380-5p	miR-380 which is highly expressed in NB repress p53-mediated apoptosis, and is associated with poor outcome in NB with MYCN amplification. miR-380 overexpression cooperates with activated HRAS oncoprotein and form tumors in mice. Inhibition of miR-380-5p in embryonic stem or NB cells results in induction of p53, and apoptotic cell death. *In vivo* delivery of a miR-380-5p antagonist decreases tumor size in NB orthotopic mouse model suggesting a promising potential therapeutic ability to miR-380 inhibitors	TH-MYCN transgenic mice of NB Orthotopic NB model	[132]
** miR-15a	miR-15a promotes the migration of NB cells through targeting the RECK-MMP-9 axis. Suppression of miR-15a decreases the migration and invasion whereas overexpression increases the migration ability of NB cell lines. These findings provide insights into the role of miR-15 in NB migration and invasion and qualify miR-15a as a potential therapeutic target.	GI-LA-N and SK-N-SH cell lines	[133]
** miR-34a	Tumor suppressive miR-34 family members targets MYCN and inhibit the growth of NB cell lines In addition, other genes also targeted by miR-34a, including E2F3, BCL2, CCND1 and CDK4 were found to be involved in cell proliferation or apoptosis Over expression of miR-34a in NB cell lines induces cell cycle arrest decreases cell number and induces apoptosis. miR-34a significantly reduces tumor growth in an *in vivo* orthotopic murine model.	Kelly, SK-N-AS cell lines NB1691luc NB cell lines NB xanograft mouse model	[135]

* Analysis in MB; ** Analysis in NB. Reviewed in [102,235].

### 9.1. miRNA Antagonists

The most common strategy to ablate miRNA function is achieved by single-stranded oligonucleotides with miRNA complementary sequences (antisense). The backbones of these are chemically modified in order to increase the affinity towards the endogenous miRNA or, alternatively, to trigger the degradation of the endogenous miRNA. Examples of miRNA antagonists are anti-miRNA oligonucleotides (AMOs), locked-nucleic acids (LNA), miRNA sponges, miRNA masking and small molecule inhibitors specific for certain miRNAs [234,239,240]. The most widely used AMO are 2¢-O-methyl AMOs, 2¢-O-methoxyethyl AMOs, and (LNA) AMOs [3,241,242,243,244]. miR-21 inhibition *in vitro* provides a good example of an anti-miR application in cancer cells. Knockdown of miR-21 suppressed cell growth and activated apoptosis in cultured glioblastoma and breast cancer cells [12,161], reduced invasion in metastatic breast cancer, lung metastasis [245], colon cancer [99], glioblastoma [246] and MB culture cells [100].

*In vivo* studies also showed that treatment with LNA–anti-miR-21 oligonucleotides disrupted glioma growth [247]. Other studies have shown that intravenous administration of AMOs against miR-16, miR-122, miR-192 and miR-194 in animals offer efficient and sustained silencing of corresponding miRNAs [248]. As an alternative to chemically modified antisense oligos, vector-encoded RNA molecules, known as “miRNA sponges” have recently been designed to contain multiple tandem binding sites to a miRNA of interest [249,250,251]. miRNA sponges function by immobilizing the target miRNAs and thus blocking their regulatory function in the cell. miRNA sponges are transcripts under the control of strong promoters (RNA polymerase II) and contain multiple tandem binding sites to a miRNA of interest. miRNA sponges are able to inhibit miRNA function as strongly as AMOs. In addition miRNA sponges have the advantage of being stably integrated into the genome, thus opening up the possibility of creating stable cell lines and transgenic animals that are functionally deficient for a specific miRNA family reviewed in [234,252,253]. However, the risk of insertional mutagenesis in target cells as well as challenges in the delivery of these non-small-molecules makes sponges not ideal for therapeutic applications. “miRNA masking” is another alternative strategy which refers to a sequence with perfect complementarity to the binding site of an endogenous miRNA in the target gene. This “miRNA masking” can form duplex with the target mRNA with higher affinity, therefore blocking the access of endogenous miRNA to its binding site without the potential side effects of mRNA degradation by AMOs [254].

The recent development of small molecules having the ability to modulate the function of miRNAs, renders miRNA pathways susceptible to small molecule inhibitors. Small molecule inhibitors have several advantages over traditional nucleic acid-based tools to manipulate miRNA function, since they are more easily delivered into humans or animals, they are intracellularly more stable, and they are less expensive to manufacture. Based on these promising developments, it can be expected that several new small molecule inhibitors of miRNA function will be discovered in the near future [254].

### 9.2. miRNA Replacement Therapy

miRNA replacement therapy is the strategy of restoring tumor suppressive miRNAs which are lost in cancer cells, to re-establish their “normal” functions. In this regard miRNA mimics are either single or double-stranded miRNA that have the same sequence as the depleted, naturally occurring miRNA. As a consequence, miRNA replacement therapy is expected to have limited off-target effects. The simplicity, specificity and potency of mimicking miRNA precursors have made this an exciting approach to target genes for both research and therapeutic purposes [239].

Several technologies have proven effective in delivering therapeutic miRNAs to tumor tissues *in vivo*. These include adenoviral or lentiviral vector-based systems that were originally developed for gene therapy. A viral approach has been used to reintroduce Let-7 expression in an orthotopic lung cancer mouse model [255]. The reintroduction of Let-7 significantly reduced lung tumor formation *in vivo* [256]. In line with this is the reintroduction of miR-26a in a liver cancer mouse model, which resulted in the inhibition of cancer cell proliferation, induction of tumor-specific apoptosis, and prevention of disease progression [257]. Another example that shows the value of miRNA replacement is provided by miR-34a [258]. In this case, therapeutic delivery of miR-34a led to an accumulation of miR-34a in the tumor tissue, suppression of known miR-34a target genes and, most importantly, inhibition of lung tumor growth [240]. Similar to the replacement therapy approach but differing in miRNA delivery, unmodified miR-145 and miR-33a have been delivered by using polyethylenimine (PEI) in a mouse model of colon carcinoma, a method that proved efficacious [259]. Likewise transient transfection with synthetic miR-16 significantly reduced cell proliferation in prostate cancer cells *in vitro* and reduced metastasis *in vivo* [260]. Another example of the success of miRNA replacement therapy is demonstrated by Garzia *et al.* [109]. The group showed that MB tumor burden can be reduced in a xenograft model by miR-199-5p overexpression, indicating the therapeutic potential of miR199b-5p. Also replacement for miR-15 and miR-16 [261], that are often deleted in CLL patients [261,262,263], reduces proliferation and triggers leukemic cell death, reviewed in [264].

Together, the evidence showing that miRNAs can function as tumor suppressors, and that the synthetic versions of these miRNAs robustly interfere with tumor growth, reflects the importance of miRNAs in cancer and strongly supports the development of miRNA mimics as ideal candidates for therapeutic intervention.

### 9.3. Challenges of miRNA-based Therapies

miRNAs biological instability, the difficulties of specific delivery, and the insufficient uptake for effective target inhibition challenge the development of miRNA-based therapies. To overcome these obstacles chemical modifications in oligonucleotides have been investigated, however the drawbacks of improved delivery to tissues after chemical modifications are impaired biological activity and increased toxicity. To solve these problems, novel miRNA formulations, including nanoparticles, polymers or viral transduction approaches, are currently under development. Moreover, effort is put into the engineering of effective systems that deliver synthetic miRNAs selectively to diseased tissues in order to limit potentially toxic off-target effects, reviewed in [265]. Finally and importantly homeostasis of the brain is dependent on the blood-brain barrier (BBB) that dictates the exchange of essential nutrients and limits the entrance of molecules and immune cells into the central nervous system. This barrier therefore forms yet another important hurdle for effective miRNAs treatment of malignant brain cancer. Hence effective brain delivery for miRNAs may require the design of particular therapeutic approaches that overcome this physiological obstruction, such as the use of nanoparticles, immunoliposomes, peptide vectors or carrier-mediated transport through the BBB, reviewed in [266].

In summary, the recent fast progress in the field has triggered some optimism about the development of successful molecules targeting microRNAs. However miRNAs targeting approaches are still at the preclinical stage and even with promising preclinical responses to molecules targeting miRNAs or their target genes, it is expected that tumor types and context will add to the complexity and heterogeneity of response to any of the above mentioned strategies. New therapies targeting miRNAs or its’ target genes may best be applied in the future together with molecular profiling of cancers for clinical stratification and selection of combination therapies.

## 10. Concluding Remarks

Within the last decade, substantial progress has been made in understanding embryonal tumor development through the discovery of deregulations in the highly conserved developmental signaling pathways. However, effective targeted therapies still remain elusive and the development of novel therapeutic strategies remains an urgent goal. Risk stratification and drug-response prediction are the central elements of targeted therapies. During recent years, research has focused mainly on gene expression profiling in embryonal tumors and a number of important studies have been published. As gene expression profiling reaches a plateau and begins to face limitations, miRNA signature is a rising star that may provide new resolutions to old problems. Numerous miRNAs are dysregulated in pediatric cancers, and evidence suggests miRNAs as attractive targets in cancer therapy. A greater understanding of miRNA biology and the development of suitable delivery systems are required to translate basic research results into clinical practice. miRNAs are much more stable in body fluids than mRNAs, raising the exciting prospect that miRNAs might be used as non-invasive biomarkers for disease monitoring and risk stratification under specific circumstances. Insights gained from miRNA studies may open a new era in pediatric brain cancer treatments which provides improved patient selection for targeted agents, and forms the basis for the development of novel therapeutics and/or early disease biomarkers.
